# Evaluating the benefit of early patient and public involvement for product development and testing with small companies

**DOI:** 10.1111/hex.13731

**Published:** 2023-02-14

**Authors:** Elanor C. Hinton, Cameron Fenwick, Martin Hall, Mike Bell, Julian P. Hamilton‐Shield, Andrew Gibson

**Affiliations:** ^1^ NIHR Bristol Biomedical Research Centre Nutrition Theme, Bristol Medical School University of Bristol Bristol UK; ^2^ Research and Enterprise Division University of Bristol Bristol UK; ^3^ Government Office for Technology Transfer Cardiff University Cardiff UK; ^4^ PPI Contributor; ^5^ NIHR Applied Research Collaboration West, Bristol Medical School University of Bristol Bristol UK; ^6^ Department of Health and Applied Sciences University of the West of England Bristol UK

**Keywords:** CUBE framework, evaluation, industry, product development, public and patient involvement

## Abstract

**Introduction:**

There is a growing understanding of the benefits of patient and public involvement (PPI), and its evaluation, in research. An online version of the CUBE PPI evaluation framework has been developed. We sought to use the CUBE to evaluate the value of early PPI with two small healthcare companies during product development.

**Methods:**

Contributors were recruited online and had lived experience of either type 1 diabetes or obesity. Two 1‐h sessions were run with a company developing a smartphone application to manage diabetes (DEE‐EM): one with young people (YP; *n* = 5) and one with parents (*n* = 7). Two 1‐h sessions were run with a company developing a weight‐loss product, both with adults (*n* = 7 in each session). Sessions were facilitated by an independent University researcher and attended by company representatives, who presented their product. One facilitator led the evaluation of the session by giving a demonstration of the CUBE and asking simple questions in the YP session.

**Results:**

A high proportion of contributors completed the CUBE (80.5% DEE‐EM; 93% Oxford Medical Products). Responses were positive to all four CUBE dimensions (in italics). Contributors felt there were diverse ways to *contribute* to the sessions, and that they had a strong *voice* to add to the discussion. Balance was achieved regarding whose concerns (public or company) led the *agenda*, and contributors felt that both companies would make *changes* based on the discussion. The supportive attitude of both companies resulted in most contributors feeling comfortable participating in PPI sessions with the industry, while recognising the profit‐making aspect of their work.

**Conclusions:**

PPI with small healthcare companies is both feasible and worthwhile. The CUBE framework facilitated the evaluation of the interaction between experts in different knowledge spaces. We provide recommendations for future projects, including considerations of who should participate and the level of implicit endorsement of the product that participation implies.

**Patient or Public Contribution:**

People with lived experience of type 1 diabetes or obesity were invited to contribute to one of four PPI sessions, which they then evaluated. One contributor agreed to contribute to the analysis of the evaluation data and interpretation and preparation of the manuscript.

## 1 INTRODUCTION

Clinical researchers and funders of such research in the United Kingdom, for example, the National Institute for Health and Care Research (NIHR) and UK Research and Innovation (UKRI), are increasingly recognising the importance and value of patient and public involvement (PPI) in all stages of the research process. This important step is also emerging in Europe, through the support of the Innovative Medicines Initiative,^1^ and also in the United States, as exemplified by the Patient‐Centred Outcomes Research Institute (PCORI; https://www.pcori.org/). Funders encourage working closely with people with lived experience of health conditions as a mechanism to enhance the translation of health research findings into societal and economic benefits. Funding schemes supporting such collaboration, particularly from NIHR, require consideration as to how patients and the public will benefit from and be involved in research.^2^

Concurrent with the rise of PPI in research is a call for greater evaluation of that PPI, to determine its value and quality.^3^, ^4^, ^5^ The process of evaluation of PPI, allows PPI contributors to consider whether they have been given the opportunity to participate in a meaningful way, which can then have a positive impact on the research study in question. Through the evaluation, researchers gain insight into the contributors' experiences of the sessions, in turn giving the opportunity to improve aspects that may be less well received. Moreover, researchers and funders need to critically consider costs, benefits, and risks, as well as how best to conduct PPI for benefits to be realised.^3^, ^6^ A range of methodologies is available to evaluate PPI in research, often chosen based on the intended outcomes and the time frame available to conduct the research.^4^ These approaches range in simplicity, from preparing an ‘impact log’ on the outcomes of the PPI, using the CUBE framework,^7^ to the more comprehensive Public Involvement Impact Assessment Framework^8^ or Realist Evaluation.^9^ The CUBE framework was chosen for this project to reflect the consideration of the differing ‘knowledge spaces’ (the conversation space in which different types of expertise from the public, healthcare providers and other professionals are shared) that are important when evaluating interactions between the public and other organisations on healthcare issues.^4^, ^10^, ^11^

In brief, the CUBE framework was developed through a combination of reviewing the theoretical literature on social inequality and practical workshops with members of the public.^7^ The framework allows the researcher to evaluate PPI across four dimensions: voice (the extent to which contributors feel they have a weak or strong voice in decision‐making); contribute (the number of ways to get involved to accommodate different contributors' needs); agenda (the balance between organisation and public contributor concerns); change (the willingness or resistance to change by the organisation). It can be used to compare the experience of PPI across different organisations.^7^ Since the publication of this framework, Gibson and colleagues in People in Health West of England have developed an online version of the CUBE evaluation, allowing greater flexibility, asynchronous input and timely responses following PPI sessions.

Companies have used market research, conducted in‐house or by external market research agencies, to garner public opinion during the medical product lifecycle (MPLC). Market research is the passive extraction of information from the public, via focus groups, individual interviews, and surveys. By contrast, PPI encourages active involvement by patients and the public in a co‐design process. It is recognised that opportunities exist for PPI throughout the MPLC and health technology assessment,^12^, ^13^ but there is a lack of consensus regarding how and when is best to engage with patient preferences.^14^, ^15^, ^16^ Health preference research often employs discrete choice experiments to assess patient preferences,^14^ however, it could be argued that alternative, discursive approaches, such as PPI, may be more appropriate at the early design phase of the MPLC. In the United States, for example, the Center for Drug Evaluation and Research has developed a programme to assist companies in ‘Patient‐Focused Drug Development’, to ‘ensure that patients' experiences, perspectives, needs and priorities are captured and meaningfully incorporated into drug development and evaluation’.^17^ In the United Kingdom, the Aims 2 Trials have set up a steering committee of representatives with autism who actively advise on drug development and trials with industry partners and researchers.^18^

The novel objectives of this project were twofold: (i) to use the CUBE framework to evaluate whether PPI sessions for small start‐up companies were acceptable and valuable from the contributors' perspective and (ii) to assess whether PPI might be beneficial for companies who are developing products for healthcare and looking to partner with NIHR Biomedical Research Centres (BRCs) to move their plans forward. A further key question posed was whether the profit‐making nature of a company changes the ethos of the PPI and therefore, whether the approach taken to PPI needs to be different with a company compared to with a research institution. Our final aim was to produce a set of recommendations on conducting PPI with small companies.

## 2 METHODS

We set out to evaluate the introduction of PPI in the MPLC with two UK‐based small technical companies. One company, DEE‐EM, was in the initial product development phase of a novel smartphone app for type 1 diabetes management (https://www.dee-em.com/). The second, Oxford Medical Products (OMP), has developed a weight‐loss product, a hydrogel in capsule form which expands in the stomach with the aim of increasing fullness and reducing food intake and were at the beginning of the validation phase, planning their first in man safety and feasibility trial (https://www.oxfordmedicalproducts.com/). These two small companies were chosen as had approached the Bristol BRC for help in gaining public opinion from patients with relevant lived experience. We worked with these two companies in differing ways: for the DEE‐EM project, only the company spokesperson and the PPI facilitator (M. B.) were actively involved in the session, whereas for the OMP project, the company spokesperson, PPI facilitator (E. C. H.) and a bariatric surgeon (chief investigator on the future trial) were involved.

### 2.1 Contributors

Contributors were people with lived experience of either type 1 diabetes (DEE‐EM sessions) or obesity (OMP sessions). Recruitment material was generated with input from each company. Advertisements were placed online with the support of People in Health West of England, Diabetes UK, Obesity UK and social media (Twitter and Facebook). For DEE‐EM, two sessions were planned: the first with young people (YP) under 18 years with type 1 diabetes and the second with parents of YP with type 1 diabetes. For OMP, two sessions were also planned, both with adults with a lived experience of obesity. We aimed to recruit between six and eight contributors for each session. Following joint guidance from the National Research Ethics Service and NIHR INVOLVE initiative,^19^ ethical approval was not sought for this project. Active involvement in PPI and its evaluation is conducted *with* the contributors as equal partners, rather than ‘to, about, or for them’,^12^ ^,p.1^ as research participants. The PPI sessions were conducted with the utmost respect and care for contributors giving them the right to take part and share the details they choose to during the sessions. All contributors gave permission for the sessions to be recorded. The scores from the CUBE evaluation are collected anonymously, and all quotes from the recordings were also rendered anonymous before inclusion in this paper.

### 2.2 Session design

The design of PPI sessions with both companies was of a similar format. A facilitator was identified (M. B. or E. C. H.) who coordinated the recruitment (see below) to two online sessions over Zoom with each company, sent invitations and circulated any premeeting information. Before each session, the facilitators helped to design slide material with the companies to share with contributors to maximise the opportunity for participation, while ensuring that the feedback would be relevant to the company's product development.

During each hour‐long session, the facilitator provided the introduction, including details of online meeting etiquette and confidentiality, and then introduced the company representative(s) and any others present on the call. Each person individually told the group what their role was in the organisation they were affiliated with, so it was clear who worked for the company, in healthcare or for the university. The company representative then introduced the product and in the case of DEE‐EM led the questions, whereas the facilitator led the questions during the OMP sessions. To finish the DEE‐EM session with YP, the facilitator (M. B.) asked some evaluation questions (see below). At the end of all sessions, one of the facilitators (E. C. H.) explained the evaluation part of the session and demonstrated the evaluation tool (the CUBE, see below). During the demonstration, the facilitator explained the meaning of each of the CUBE questions and contributors were able to ask questions if further clarification was needed. The facilitator sent an email to each contributor (or their parent in the case of the YP DEE‐EM session) afterwards with the details of how to evaluate the session if they wished. Contributors were provided with an honorarium to participate in sessions, based on the PPI rate advocated by the NIHR.^20^

### 2.3 Evaluation tools

For the DEE‐EM session with YP, following the discussions regarding the product, a series of brief questions were asked, as follows: (1) Did you find it interesting? (2) Do you feel listened to? (3) Was it fun? (4) What would have made it better? (5) Is there anything else you want to say about this topic? To ensure the anonymity of the responses, all names on the Zoom call were changed to ‘Me’, then contributors could respond with a number from 5 to 1 in relation to each question (e.g., 5 for very positive, 1 for very negative). These were recorded in the meeting chat and saved by the facilitator.

**Figure 1:**
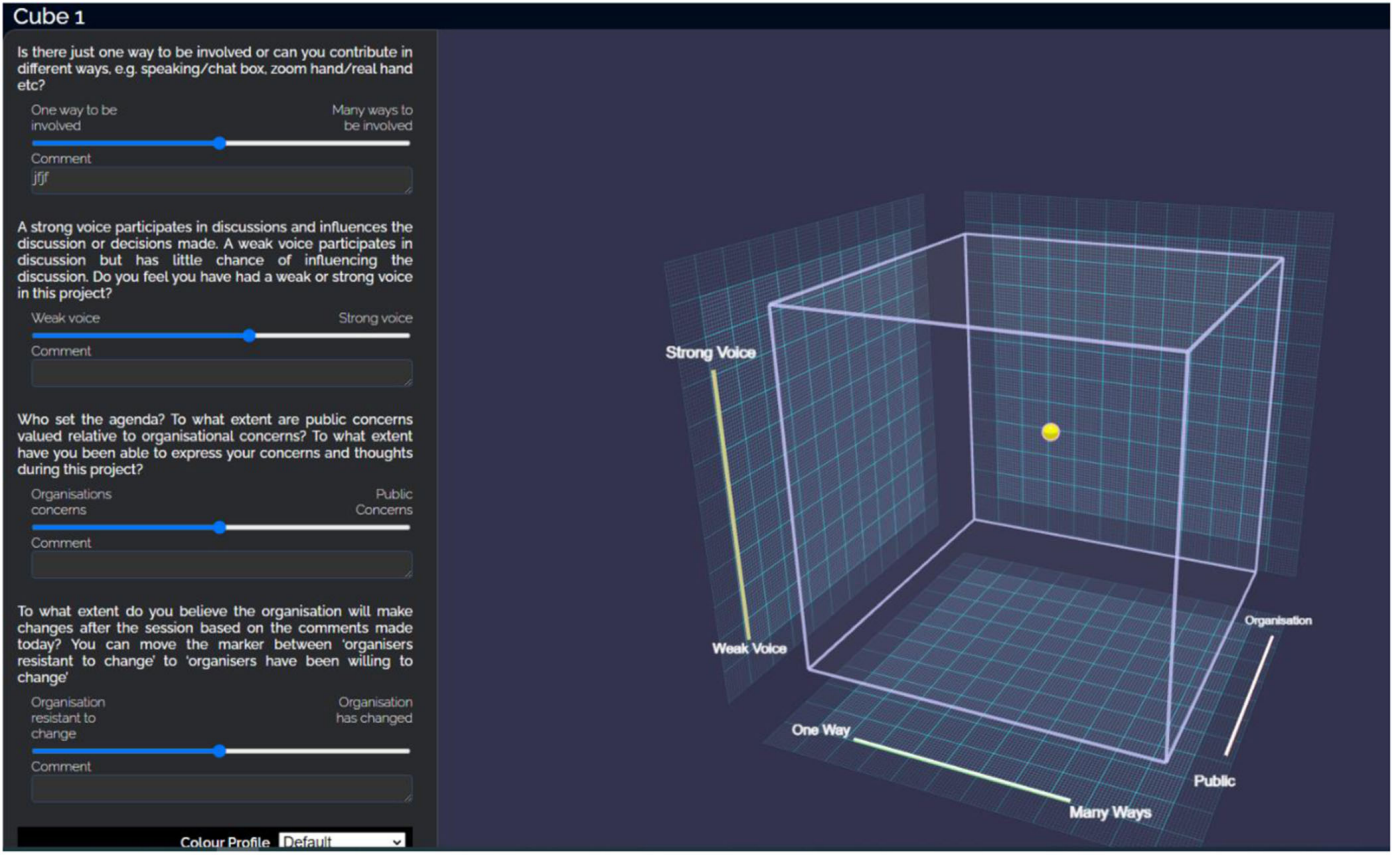
Screenshot of the CUBE. Questions for each dimension appear on the left of the screen with the slider to move the data point in blue. A comment box is provided under each question. The CUBE appears on the right, with each dimension on a different axis: Contribute on the *x*‐axis, Voice on the *y*‐axis, agenda on the *z*‐axis and change as colour of the data point (yellow is neutral; red is resists change, green is willing to change). The cube can be rotated using the mouse so that contributors can explore the data points in relation to the different dimensions.

For all four sessions, contributors were asked to fill in the online version of the CUBE framework.^7^ The four questions, one relating to each dimension, were adapted for this project (Table 1). Contributors answered each of the questions by using a slider under the question from 0 to 1 on each dimension. This creates a data point on the CUBE, which moves in three dimensions and changes colour according to the final question on organisational change (Figure 1 for an example of the CUBE). A comment box appeared for each question so that contributors could add additional details on their experience in relation to each dimension. The scores and comments were recorded anonymously in a spreadsheet associated with the CUBE link. The mapping of responses on the CUBE could be viewed by all contributors (after logging into the online system).

**Table 1:** CUBE questions.

CUBE dimension	Question
Contribute	During this project, have there been different ways that have enabled you to contribute to the discussion?
Voice	A strong voice participates in discussions and influences the discussion or decisions made. A weak voice participates in the discussion but has little chance of influencing the discussion. Do you feel you have had a weak or strong voice in this project?
Agenda	Who sets the agenda? To what extent have you been able to express your concerns and thoughts during this project? Have the concerns of the organisers of the project dominated? You can move the marker between ‘organisers concerns’ and ‘public concerns’ and again add notes if you wish.
Change	To what extent do you believe the organisation will make changes after the session based on the comments made today? You can move the marker between ‘organisers resistant to change’ to ‘organisers have been willing to change’.

Finally, contributors were asked an additional question: ‘How did you feel during the session about contributing to the development of a product by a company rather than a university or healthcare provider?’

### 2.4 Analysis plan

Scores from the questions given to the YP were summarised, and the scores on the CUBE for each session were collated and summarised by the median and interquartile range (IQR; due to the ordinal nature of the data). Comments made by contributors using the CUBE, and responses to the additional question, were collated into a table to summarise their experiences of each session and narratively synthesised across common themes between the sessions.

## 3 RESULTS

The DEE‐EM sessions took place early evenings of May 2021, and the OMP sessions took place in July 2021.

### 3.1 Contributors

#### 3.1.1 DEE‐EM YP session

Four YP with type 1 diabetes joined the call, with one parent in attendance in support of one of the YP.

#### 3.1.2 DEE‐EM parent session

Seven parents joined the second call the following day.

OMP session 1: Seven contributors took part.
OMP session 2: Seven contributors took part (different people from OMP session 1).

### 3.2 Evaluation of DEE‐EM YP session

Contributors to the DEE‐EM YP session (1) were positive about their experiences during the session. When asked if they found the session interesting, three out of four contributors gave the highest score (5/5), with one response of 4/5. When asked if they felt listened to, all four contributors gave the highest score of 5/5. All four reported that they had enjoyed the session, with quotes such as ‘I would love to do it again’ and ‘yes, I enjoyed it, it was interesting learning about it before it [the app] was out’. When asked what would have made it better, all four said they would have preferred the session to have been held in person; however, two of the contributors clarified that this was because they could miss school. Finally, when asked if there was anything else they wanted to say about the app, the contributors made useful suggestions: ‘An advice section possibly?’ [further clarification was that this was advice from doctors to the app users]; ‘Being able to upload it to our doctors’; ‘It looks amazing how it is already’. The parent who attended the DEE‐EM YP session felt it had been an interesting and informative session: ‘I think (as far as I can tell with a teenager) she really enjoyed the event. And I like anything that lets her pause to think about what she needs to better manage her condition’.

### 3.3 Summary of the CUBE results

**Figure 2:**
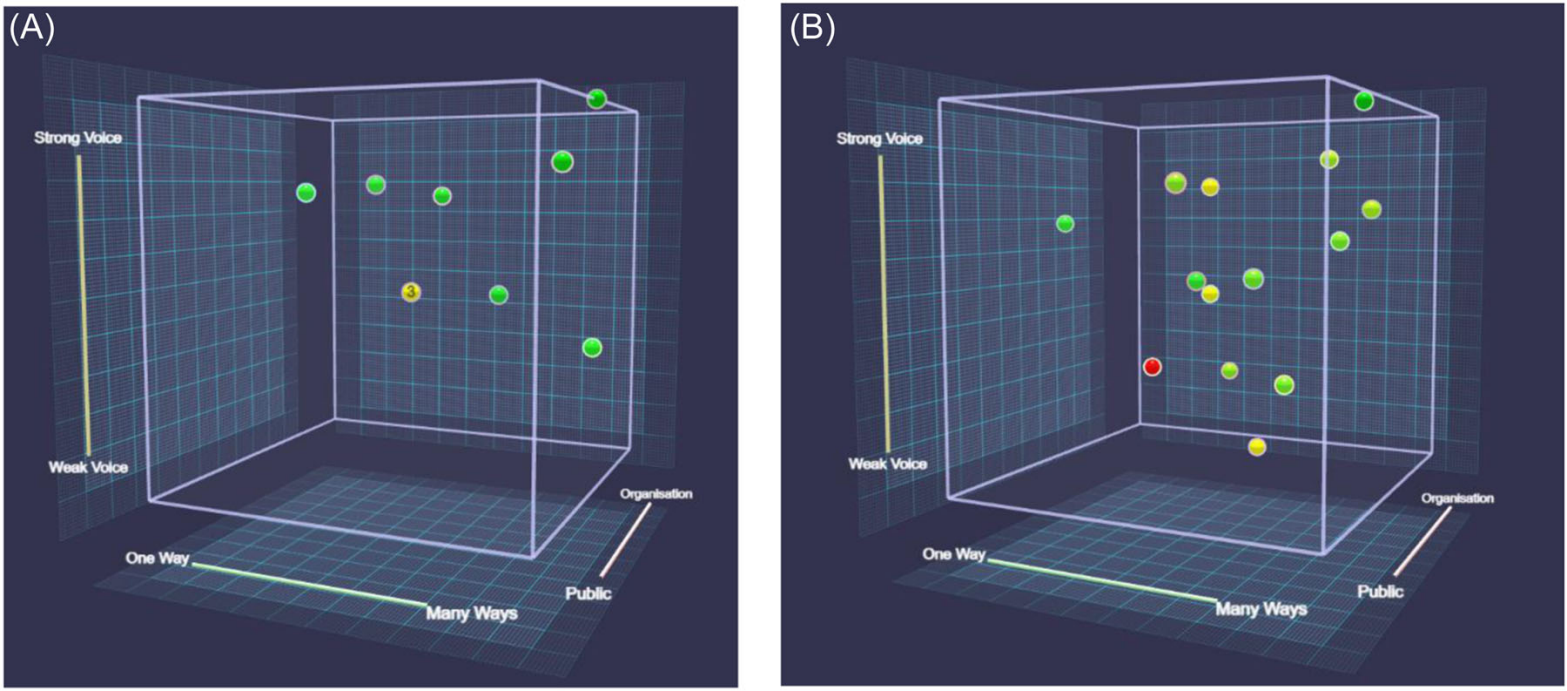
(A) Final CUBE with contributor's responses for DEE‐EM; (B) final CUBE with contributor's responses for OMP. The CUBE appears on the right, with each dimension on a different axis: Contribute on the *x*‐axis, Voice on the *y*‐axis, agenda on the *z*‐axis and change as colour of the data point (yellow is neutral; red is resists change and green is willing to change). The cube can be rotated using the mouse so that contributors can explore the data points in relation to the different dimensions.

**Figure 3:**
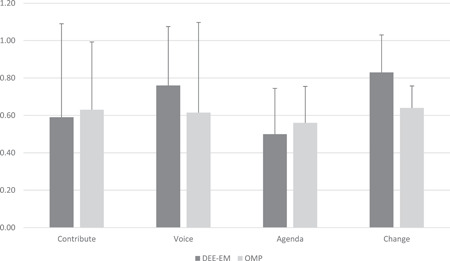
Median (IQR error bars) scores on each of the CUBE evaluation dimensions over the two sessions held for each company. IQR, interquartile range; OMP, Oxford Medical Products.

A high proportion of the contributors did provide an evaluation of the session they attended using the CUBE: 75% from the DEE‐EM YP session; 86% from the DEE‐EM parent session; 93% of the adults who attended the OMP sessions. Figure 2 depicts the final CUBEs for each company. Figure 3 depicts the median values and IQR error bars for each of the four CUBE dimensions (see Table 1 for question details).

### 3.4 CUBE dimension: Contribute

The mean scores in Figure 3 and data points in Figure 2 show that contributors reported that there were several different ways that enabled them to participate in the discussion (Table 2, contribute dimension), as well as the evaluation afterwards. One contributor used this opportunity to suggest other ways that could be incorporated into such sessions: ‘Consideration could be given to surveys and questions too’ (DEE‐EM session 2). Another contributor was not clear on the meaning of this question: ‘I wasn't really sure what this question meant. I have answered based on the fact that I have been involved in two ways i.e. zoom meeting and now this cube follow‐up. Hope I have understood correctly’. A greater explanation of the CUBE in advance might provide more clarity on the intended meaning of the questions.

**Table 2:** CUBE dimensions quote from the sessions with DEE‐EM and OMP.

CUBE dimension	DEE‐EM sessions	OMP sessions
Contribute	‘We all made good points so I think they will take that into count’.	‘By using chat was good as you can't always get your view over so using chat is easy’
	‘We were able to speak and to write in the chat box, which was very useful when being interrupted by an excited 3 year old!’	‘Lots of questions were asked’
	‘You could click on the raise hand button or type in what you wanted to say’.	‘I really enjoyed the mixture of engaging techniques used. Such as video, polls, chat box, contributing with the camera on’
	‘Chat feature on zoom, zoom discussion, initial thoughts over email’.	‘Zoom hand and my hand’
		‘I thought the fact we could talk or write in the chat was great. If we weren't in a pandemic, I have no doubt a face to face option would be offered’
		‘So far, only discussion has been via the zoom meeting’
Voice	‘No one was ignored what shows that they listened’.	‘In the middle I think as I'm always prepared to hear others views’
	‘I feel that my voice, thoughts and ideas were heard and valued’.	‘I felt listened to’
	‘Felt that the recommendations were listened to and understood and considered for future developments to the app’.	‘I felt very comfortable to contribute and the environment was very safe to contribute. It was great to see everyone who wanted to contribute to the discussion had the opportunity’
	‘Lots of valid points were made by all speakers. We all have a lot of shared opinions and ideas so it was important to listen as well as discuss, which the group seemed to do well, in my opinion’.	‘Strong as I said what I needed to say’
	‘I am more vocal during face‐to‐face meetings’.	‘I felt my voice was strong and I was heard, but I feel the same about the others also talking. Potentially, the people using the chat had a weaker voice’
		‘I made various points and felt free to do so’
		‘I felt able to participate wherever possible, notwithstanding time constraints’
Agenda	‘In the meeting we were encouraged to voice any concerns that we might have’.	‘No concerns as it went really well’
	‘I feel that it was equal and everyone was given their chance to voice their concerns and opinions’.	‘I think public concerns prevailed’
		‘Very open dialogue between all parties’
	‘Lots of opportunities to discuss. Ideas and suggestions seemed to be taken on board’.	‘It was balanced’
	‘I think because of his circumstances, the organiser's concerns are also public concerns’.	‘I felt it was a good half and half, the organisation definitely has their concerns but the publics were addressed as and when we got there. We were kept very well informed’
		‘I felt that the organisers were genuinely interested in our views, though not sure whether financial considerations will eventually outweigh “ideal” product’
		‘I felt the meeting was very much about the organisers making a great effort to discover public feelings and concerns’
Change	‘They wouldn't make the meeting for no reason’.	‘Hopefully that our input has helped the research’
	‘I definitely feel that our thoughts were valued and that the ideas we had for the app would be considered as we were a good cross section of parents with different aged children using different kinds of technology so listening to our thoughts would allow the product to be useful to so many who have the same worries and issues that we have’.	‘Time of course will tell. However, in the meeting there was a sense of being listened to as a group’
	‘The organisers were very interested in our opinions and ideas to improve the app. I am fully confident that if the ideas we had can be done then they will be added to the app’.	‘I feel the organisation will have definitely taken the publics thoughts and opinions on and therefore will change what they can to do what they can to address our comments’
	‘Recognise its early days and adaptations and improvements can take time’.	‘I felt organisers were genuinely interested in our views and I would like to think that theses will be taken fully into account in the design and launch of the product’
	‘The researchers were very open to suggestions‐ listened to what was asked and what was wanted and seemed to understand why different things were asked ‐ there were very few no's throughout!’	‘I felt that necessary changes to the research would be made if necessary but have no real way of knowing for sure of course’

Abbreviation: OMP, Oxford Medical Products.

### 3.5 CUBE dimension: Voice

The scores in Figure 3 and quotes in Table 2 show that contributors to all the sessions felt listened to and had ample opportunity to get their ‘voice’ heard in different ways. The quotes in the table suggest the format of the online session may have influenced the extent to which some people felt able to contribute.

### 3.6 CUBE dimension: Agenda

The comments provided by contributors on the extent to which company or public concerns dominated the session suggest that a good balance was achieved during sessions with both companies (Table 2, agenda dimension), with comments such as ‘Very open dialogue between all parties’. The company representative from DEE‐EM had personal experience with type 1 diabetes, and this was acknowledged in this section by one contributor: ‘I think because of his circumstances, the organiser's concerns are also public concerns’. However, with scores close to the centre of the agenda dimension (see Figures 2 and 3), both companies achieved a good balance between their specific concerns and allowing the public contributors to bring their concerns into the discussion: ‘I felt it was a good half and half, the organisation definitely has their concerns, but the publics were addressed as and when we got there…’

### 3.7 CUBE dimension: Change

The final CUBE dimension captures the extent to which the contributors believed the organisation would make changes after the session based on the comments by the public made during the session. As Figure 3 shows, contributors gave moderately high scores towards the ‘organisation have been willing to change’ rather than resistant to change. The DEE‐EM scores were particularly high on this dimension, again perhaps reflecting the emotional investment the company's representative has in developing the app for diabetes management. There was also a sense that for both companies, the sessions had been held early in the process, and that over time, it would become apparent if concerns had been addressed going forward, although one contributor expressed uncertainty regarding how they would find out (Table 2).

### 3.8 Contributing to PPI for industry

The final question asked attendees to consider how they felt about contributing to the development of a product by a company rather than a university or healthcare provider. Two contributors to the DEE‐EM sessions and nine contributors to the OMP sessions answered this question. Overall, these comments were positive (‘I felt rather informed and comfortable…’ OMP), with one person commenting that ‘I am not sure that it makes much difference who supplies the product’ (OMP), although one contributor commented ‘The fact that the product is being developed by a company made me slightly more uncomfortable than if it had been a university or health provider. I do not have the same level of trust in private companies’ (OMP). However, the focus by both companies on making improvements to healthcare that would be valued by the public was appreciated and reflected in the comments: (i) As reflected in the change scores above, the more personal emphasis in the DEE‐EM session was valued (‘I feel very positive about contributing to the development of the app by a private company. I think this is possibly due to the personal link by the developer to type 1 and that he is doing this with true understanding of the issues that type 1's deal with not only daily but every minute of the day!’ DEE‐EM); (ii) The supportive environment in the OMP session was also appreciated: ‘I felt very comfortable in the session. It was a very supportive environment and great to meet specialists in the field. More than happy to contribute to a company product. There was a sense of a caring attitude from the company representatives, and they certainly made me feel at ease and comfortable to talk about not only the product but also about my individual issues with weight loss’ (OMP).

The financial or profit‐making aspect of working with industry was reflected in comments from the sessions with both companies. One contributor referred to the need for private investment to develop new products: ‘Funding for health is so restricted that I expect most health innovations to be led by private companies with a personal goal’ (DEE‐EM). Another contributor agreed that external funding was necessary but made them feel slightly less comfortable: ‘I personally would always feel better if profits are not involved but I recognise that is not always possible. The team seems well‐respected in their fields, appropriately qualified and I haven't seen or heard anything that has caused me alarm’ (OMP). Others remarked on the need for companies to make a profit, but that did not influence their willingness to contribute: ‘I felt ok contributing to research by a company, but it does make me wonder how much are those running the company going to profit?’ (OMP) and ‘I am fully aware that the purpose of this research is ultimately the production of a product for financial gain. OMP would not be bothering with this research if it did not believe that there is money to be made in the long run. This does not impact on my willingness to be involved, as long as the end product is medically safe, healthy, effective in achieving weight loss, ethically and morally promoted, and supported and financially affordable’ (OMP). One of the contributors felt guarded coming into the session with OMP but felt differently after the session: ‘I felt I needed more information about the product to be able to trust them. After listening to the presentation, I felt more positive and open to the idea that also a company could provide a product that would be effective in improving public health’.

Addressing our final research question, the different formats of the sessions, in terms of who was present and facilitating the sessions, may have influenced the responses to the question regarding contributing to PPI for the industry. Some contributors with lived experience of obesity who attended the OMP session had also attended another PPI session for a different project with the facilitator (E. C. H.) earlier in 2021. Some comments suggested that greater trust or endorsement of the product and company may have been given as the university researcher was known to some of the contributors: ‘I felt fine with the research coming directly from you’. Indeed, there may have been some ambiguity as to the extent of involvement between the company, university and clinicians involved in the OMP sessions, when answering this question: ‘I think I was somewhat more guarded and sceptical generally about contributing towards the development of a product of a company rather than a University or Health care provider (although, to the uninitiated, this was a bit ambiguous anyway, due to the academic groups involved and mentioned) because of the obvious commercial connotations but it was reassuring, to some extent, to know that practising surgeons were involved’.

## 4 DISCUSSION

The primary aims of this project were to evaluate the use of the novel CUBE framework to assess whether PPI sessions for two UK‐based small technological companies were acceptable and valuable from the contributor's perspective and to assess whether such PPI might be beneficial for those small companies who are developing products to improve healthcare. First, this was a feasible undertaking with sufficient people with relevant lived experience willing to contribute to the sessions and importantly a high proportion of those who attended the sessions completing the evaluation. Overall, contributors to all four of the sessions gave a positive evaluation, and the majority felt comfortable interacting with representatives from ‘profit‐making’ companies. This may be explained by the caring and supportive attitude evidenced by both DEE‐EM and OMP, acknowledged by contributors during their sessions, and potentially also the implicit endorsement by the university. Both companies expressed how valuable and informative the sessions were, and in the case of OMP, made improvements to their plans based on the active involvement of public contributors.

Mapping the evaluation of the four dimensions of the CUBE framework allowed an in‐depth exploration of the views of the contributors.^7^ Contributors felt that there were several ways that they could voice their opinions during and after the sessions. As this research was conducted during the COVID‐19 pandemic, it was required to be held online. This provided the advantage that volunteers joined from all over the United Kingdom, ensuring a breadth of opinions and responses from people of different ages, gender and lived experiences. The online environment allowed volunteers to choose the format of joining (cameras on or off, commenting directly or just via the chat). This was seen as positive by some, but others questioned whether everyone got an equal voice. While every effort was made to ensure all contributors had the opportunity and means to voice their opinions, facilitators of any future online sessions should be mindful of this issue.

While both companies had prepared slides to share with contributors with their questions, the contributors still felt there was ample opportunity for their concerns to be listened to, such that the balance of the ‘agenda’, was equitable between the contributors and the company. Contributors largely felt that the organisations were willing to change based on the PPI sessions. This is a positive result considering each volunteer only attended one session with the company, suggesting that the companies were clear in their intentions to improve their products on the basis of the opinions during the sessions. In the case of OMP, two further PPI sessions have been conducted (January 2022), with many of the same contributors returning, to contribute to the design of the first‐in‐human trial of the product and associated patient‐facing documentation. This gave an opportunity to share the positive changes made based on the initial PPI sessions, which were well‐received.

This evaluation has shown both benefits and limitations of the novel approach taken. The benefits have been to promote ‘co‐production’ of novel technology in the form of a smartphone app and to gain valuable opinions of a weight‐loss product, both of which will now be in a stronger position to meet user needs. Careful consideration was given to the choice of contributors to ensure they were specific to each companies' requirements, in terms of having lived experience with the relevant health issue. One limitation may be that the presence of the research organisation may have endorsed the product/company such that contributors may have felt a greater positive bias. However, the research organisation is unlikely to collaborate with a company they do not think is in the public interest and is more likely to prioritise the interests of patients over those of the company. Greater clarity on this issue could be given by a statement for projects that are co‐designed, that facilitation by the research organisation does not necessarily equal endorsement of the product. However, there is no reason that companies could not benefit from the recommendations from this project to conduct their own ‘in‐house’ PPI, as long as the balance of interests between public contributors and companies was maintained. In our experience, the presence of an independent facilitator helped to balance these interests, as demonstrated through the CUBE evaluation. In the growing field of PPI evaluation, published instances of the CUBE are increasing on varied topics^7^, ^21^, ^22^, ^23^; it is, therefore, difficult to compare the results of these industry‐linked PPI sessions with a specific health issue or research‐driven sessions. Indeed, as PPI is highly context‐dependent,^4^, ^6^ it is arguable whether this would be a meaningful comparison. A further limitation was the small number of young contributors to the DEE‐EM session. Adolescents living with chronic conditions may be less inclined to join PPI groups, as they are at an age when they are developing their own self‐image and avoiding stigmatisation associated with chronic illness.^24^ By developing relationships with relevant charities and clinicians, we will widen the reach of our work to include more YP with chronic conditions.

From the companies' perspectives, both voiced clear appreciation of the value added to their product development from the sessions—the sessions were not considered merely as a tick‐box exercise.^25^ Discussion has continued regarding facilitating similar sessions with different target groups who may benefit from the product for a slightly different healthcare issue or perspective (e.g., weight loss product from OMP for those with obesity‐related liver disease). Also, consideration of the appropriateness of the use of nondisclosure agreements has been discussed for future work, where PPI sessions are conducted with confidential information before the launch of a product.

Several recommendations for the future use of PPI with small companies have emerged from this work, consideration of which may benefit future, similar endeavours. These are as follows:

1. Before the session, ensure the company has clear goals for the PPI session.
2. Consider which experts are required to be part of the session: public contributors, industry, clinical and university representatives. Be mindful of how involvement in such a session may give an implicit endorsement of the company.
3. Provide clarity and support early in the session to allow contributors to have the knowledge and understanding of how they are being asked to contribute.^22^
4. Provide a demonstration and explanation of the CUBE questions before completion by contributors, to ensure that all public contributors are interpreting the question in the same way.
5. Prime the facilitator to ensure that all contributors have the opportunity to have their say during the session, be that with cameras on or off, speaking or through the chat function.
6. Complete the CUBE during the session to share the results, in keeping with an emerging emphasis on ‘practical workshops for knowledge creation’.^5^ If time does not allow this part of the process, we suggest providing the CUBE link immediately after the session while contributors are keen to give their evaluation of the session.
7. When interpreting the CUBE, consider that a higher score on a dimension may not be a better score; for example, a balance is optimum for the ‘Agenda’ dimension.
8. Ensure means to share the impact and outcomes of the PPI session, and the given contributions are planned and implemented when the novel healthcare products are tested and brought to market.
9. Use the evaluation by contributors to improve how future sessions are organised and run.

## 5 CONCLUSIONS

This project has demonstrated the feasibility and high quality of PPI sessions involving lay contributors with lived experience of a health issue relevant to novel product development with small medical technology companies. The CUBE framework successfully facilitated the evaluation of interactions between experts in the different knowledge spaces, including the lay contributors (experts in their own experience of the health issue), healthcare providers and industry professionals. Building stronger relationships between healthcare companies, patient organisations (often funded by the former^26^) and patients and the public, by following published guidelines^13^ and recommendations such as those presented here, should be a priority.^16^ The positive impact of the contributions from the public to product development, reported by both companies in this evaluation, suggests that this is a fruitful application of PPI for the future.

## AUTHOR CONTRIBUTIONS

Cameron Fenwick, Julian P. Hamilton‐Shield, and Elanor C. Hinton devised the work. Elanor C. Hinton, Cameron Fenwick, and Mike Bell conducted the PPI sessions. Elanor C. Hinton conducted the data analysis and wrote the paper. Andrew Gibson advised on the use of the CUBE. Martin Hall participated in one of the PPI sessions and reviewed the manuscript from a lay contributor perspective. All authors commented and agreed on the final manuscript.

## CONFLICTS OF INTEREST STATEMENT

Since the project reported here was conducted from April to July 2021, Dr Elanor C. Hinton was awarded a BBSRC FTMA fellowship to gain experience working with Oxford Medical Products from January to March 2022. As a result, Dr Hinton has been appointed part‐time to work directly with OMP, starting December 2022. Please note the work reported in this paper was conducted fully independently from both companies, with the support of a public coauthor, clinicians, and PPI experts. The remaining authors declare no conflict of interest.

## ETHICS STATEMENT

As per the NRES/NIHR Involve Statement (12), ethical approval was not required for this patient and public involvement piece. Taken from this statement, ʻethical approval is not needed for the active involvement element of the research, (even when people are recruited via the NHS), where people are involved in planning or advising on research e.g. helping to develop a protocol, questionnaire or information sheet, member of the advisory group, or co‐applicant’.

## Data Availability

All data generated or analysed during this work are included in this published article.
